# Prognosis of Metaplastic Breast Cancer: A Population‐Based Matched Cohort Study

**DOI:** 10.1002/cam4.71570

**Published:** 2026-01-28

**Authors:** Marie Bergman, Aglaia Schiza, Ceren Boyaci, Balazs Acs, Alexios Matikas, Johan Hartman, Antonis Valachis

**Affiliations:** ^1^ Department of Oncology Central Hospital Karlstad Sweden; ^2^ Institution for Medical Sciences, Faculty of Medicine and Health Örebro University Örebro Sweden; ^3^ Department of Oncology Uppsala University Hospital Uppsala Sweden; ^4^ Department of Clinical Pathology and Cancer Diagnostics Karolinska University Hospital Stockholm Sweden; ^5^ Department of Oncology‐Pathology Karolinska Institutet Stockholm Sweden; ^6^ Department of Oncology/Pathology, Karolinska Institute, and Breast Center Karolinska University Hospital Stockholm Sweden; ^7^ Department of Oncology, Faculty of Medicine and Health Örebro University Hospital, Örebro University Örebro Sweden

**Keywords:** metaplastic breast cancer, prognosis, retrospective, survival

## Abstract

**Purpose:**

Metaplastic breast cancer (metBC) is a rare subtype of breast cancer known for its challenging management. The impact of this histological subtype on prognosis remains unclear.

**Methods:**

Data were collected from the Swedish Cancer Registry between 2008 and 2018. Patients with metBC were matched 2:1 with breast cancer cases of no special type (NST). Survival outcomes were analyzed using cause‐specific hazard models for breast cancer specific survival (BCSS) and Cox proportional‐hazards models for overall survival (OS).

**Results:**

In total, 127 metBC patients were matched 2:1 with 245 NST patients, with a median follow‐up period of 54 months. When adjusted for matching variables and treatment‐related characteristics, metBC was not significantly associated with either BCSS (cause‐specific Hazard Ratio (HR): 1.13; 95% Confidence Interval (CI): 0.66–1.92) or OS (HR: 1.23; 95% CI: 0.83–1.82).

**Conclusion:**

With appropriate treatment, metBC may have survival outcomes comparable to NST. Larger studies with longer follow‐up are needed to provide further insights.

## 1 Introduction

Metaplastic breast cancer (metBC) is a heterogeneous group of breast cancer characterized by differentiation of the neoplastic epithelium towards squamous cells and/or mesenchymal‐looking elements [1]. It is often classified as triple‐negative disease and has traditionally been associated with worse prognosis compared to invasive breast cancer of no special type (NST) [1, 2, 3].

The higher risk for recurrence and death due to metBC observed in previous observational studies has been attributed to tumor aggressiveness per se but also to higher resistance to oncological therapy [1, 2, 3]. In recent years, studies based on more contemporary cohorts have shown a positive association between the use of chemotherapy and radiotherapy and improved prognosis among patients with metBC [4, 5]. This suggests that modern treatment strategies, such as dose‐dense chemotherapy schemes, taxane‐ and/or anthracycline‐based regimens, and radiotherapy delivered with more advanced techniques may positively influence the overall prognosis of this disease [5, 6, 7, 8, 9].

In this population‐based matched cohort study, we investigated the prognosis of patients with metBC compared to a matched cohort of patients with NST among patients treated with modern oncological therapy.

## 2 Methods

### 2.1 Study Population and Data Source

In this population‐based matched cohort study, all patients with metBC were identified through the Swedish Cancer Registry for the period 2008–2018. Patients with de novo metastatic metBC were excluded. This cohort was then linked to the Swedish National Quality Registry for breast cancer. Each metBC patient was matched with two patients with NST, using the following matching criteria: age at diagnosis (±5 years), year of diagnosis, cancer stage, and breast cancer subtype. A 2:1 matching ratio was chosen to increase statistical power and precision by maximizing the number of available patients for comparisons without substantially increasing the risk of imbalance. The matching criteria were selected to minimize confounding by factors known to strongly influence prognosis and treatment strategies, thereby ensuring a more valid comparison between patients with metBC and those with NST. Of note, nine cases of metBC were matched with only one patient with NST. The final cohort, including both cases and controls, was then linked to the Cause of Death Registry (Figure S1).

### 2.2 Study Outcomes and Definitions

All cases with metBC were matched for clinicopathologic features among patients without metaplastic breast cancer (NST) to generate comparable cohorts. The primary outcomes of interest were the association between metaplastic histology and breast cancer specific survival and overall survival probability.

Overall survival (OS) was calculated from the date of the diagnosis of metBC or NST to the date of death or last follow‐up. Breast cancer specific survival (BCSS) was defined as the time from diagnosis of metBC or NST to death from breast cancer. The Swedish definition of triple‐negative breast cancer (TNBC) differs from the international standard by also including tumors with low expression of the estrogen receptor (ER 1%–9%). In addition, the tumors must be progesterone receptor‐negative (< 10%) and human epidermal growth factor receptor 2 (HER2)–negative (immunohistochemistry score of 0 or 1+ or score of 2+ with negative in situ hybridization test results). Subtyping into luminal‐like, HER2‐positive, and TNBC groups was performed according to immunohistochemistry‐based criteria.

### 2.3 Statistical Analysis

Demographic, disease‐related, and treatment‐related characteristics were summarized using median and interquartile range (IQR) for continuous variables and counts for categorical variables. Kaplan–Meier curves were generated to assess and visualize the differences in overall survival between metBC and NST. Multivariate analyses were conducted using a Cox proportional‐hazards model to evaluate the individual effects of matching variables and treatment‐related factors on overall survival. The cumulative incidence function was applied to visualize breast cancer‐specific mortality between matched patients with metBC and NST. A cause‐specific hazard model was used to account for the competing risk of death from other (non‐cancer) causes. All statistical analyses were conducted using SPSS (version 28.0).

## 3 Results

### 3.1 Patient Characteristics

In total, 127 eligible patients with metBC and available information about clinicopathological parameters, treatment, and survival data were identified. These patients were matched with 1:2 to 245 breast cancer patients with NST. Key demographic, clinicopathologic features, and treatment‐related parameters are presented in Table 1.

**TABLE 1:** Patient, disease, and treatment characteristics of patients with metaplastic and invasive ductal breast cancer matched for age, year of diagnosis, stage, and breast cancer subtype.

Characteristic	Metaplastic breast cancer (*N* = 127)	Invasive ductal breast cancer (*N* = 245)	*p*
*Matching variables*			
Age in years, median (IQR)	67 (55–80)	65 (55–77)	NC
Year of diagnosis			NC
2008–2010	14 (11.0)	31 (12.7)	
2011–2014	55 (43.4)	103 (42.0)	
2015–2018	58 (45.7)	111 (45.3)	
Stage			NC
I	41 (32.2)	88 (35.9)	
II	71 (55.9)	140 (57.1)	
III	15 (11.8)	17 (6.9)	
IHC‐based subtype			NC
Luminal‐like	11 (8.7)	22 (9.0)	
HER2‐positive	7 (5.5)	13 (5.3)	
Triple negative	109 (85.8)	210 (85.7)	
*Disease‐related characteristics*			
Pathological tumor size in mm (IQR)	27 (15–35)	18 (11–30)	0.001
Missing	14	15	
pN status			< 0.001
N0	94 (74.0)	99 (40.4)	
N1	15 (11.8)	112 (45.7)	
N2	3 (2.4)	16 (6.5)	
N3	1 (0.8)	9 (3.7)	
Missing	14	9	
Tumor grade			0.673
I‐II	25 (19.7)	54 (22.0)	
III	74 (58.3)	142 (60.0)	
Missing	28	49	
Ki‐67 in % (IQR)	56 (34–80)	60 (30–80)	0.996
Missing	48	105	
*Treatment‐related characteristics*			
Surgery			0.241
Breast conserving	51 (40.2)	114 (46.5)	
Mastectomy	76 (59.8)	131 (53.5)	
Treatment setting			0.584
Neoadjuvant	17 (13.4)	38 (15.5)	
Adjuvant	110 (86.6)	207 (84.5)	
Radiotherapy	63 (49.6)	167 (68.2)	< 0.001
Chemotherapy (any setting)	77 (60.6)	147 (62.9)	0.906
Endocrine therapy	10 (7.9)	22 (9.0)	0.718
Anti‐HER2 treatment	6 (4.7)	13 (5.3)	0.804
Follow‐up in months, median (IQR)	48 (26–79)	60 (34.5–88)	0.004

Abbreviations: IHC, immunohistochemistry; IQR, interquartile range; NC, not calculated.

The median age was similar between the two matched cohorts. Triple‐negative breast cancer was the most predominant breast cancer subtype among patients with metBC and consequently among their matched counterparts. In terms of treatment strategies, radiotherapy was more often given in the NST group compared to patients with metBC (68.2% vs. 49.6%, respectively; *p* < 0.001) whereas no differences in other treatment strategies were observed including type of surgery in the breast (*p* = 0.241), chemotherapy at any setting (*p* = 0.906), endocrine therapy (*p* = 0.718), or anti‐HER treatment (*p* = 0.804).

### 3.2 Breast Cancer Specific and Overall Survival

The median follow‐up time was 48 months (IQR, 26–79 months), and 60 months (IQR, 34.5–88 months) for metBC and NST, respectively.

**FIGURE 1:**
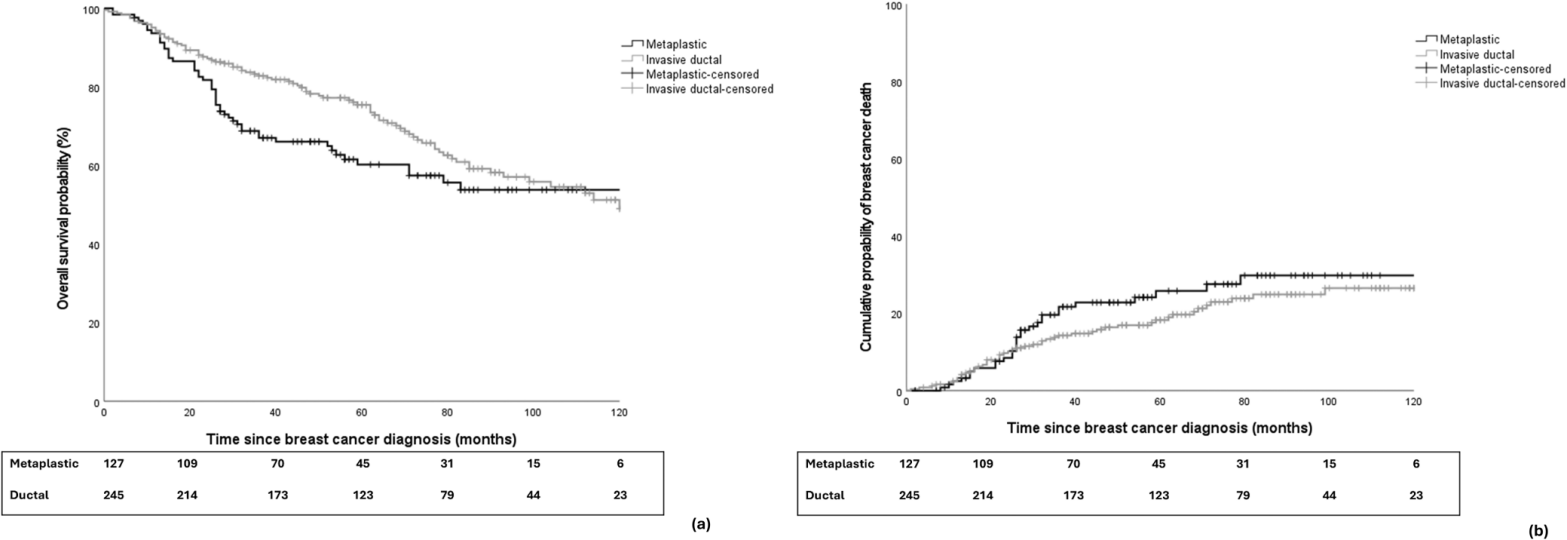
(a) Kaplan‐Meiers curves for overall survival between matched patients with metaplastic and invasive ductal breast cancer; (b) Cumulative incidence of breast cancer death between matched patients with metaplastic and invasive ductal breast cancer.

The 5‐year cumulative incidence of breast cancer mortality was 26% (95% CI: 22%–30%) for metBC and 18% (95% CI: 15%–22%) for NST, respectively (Figure 1b). This numerical difference did not reach statistical significance either in unadjusted cause‐specific Cox regression model (hazard ratio (HR): 1.28; 95% CI: 0.81–2.02) or in adjusted analyses (HR: 1.18; 95% CI: 0.71–1.97 after adjusting for matching variables; HR: 1.13; 95% CI: 0.66–1.92 after additional adjustments for treatment‐related characteristics).

Regarding OS, the 5‐year OS rate was 60% (95% CI: 55%–65%) for metBC and 75% (95% CI: 71%–78%) for NST, respectively (Figure 1a). Similarly to the analysis of BCSS, no statistically significant results were observed in Cox regression models for OS (unadjusted HR: 1.34; 95% CI: 0.95–1.90; HR: 1.25; 95% CI: 0.84–1.97 after adjustment for matching variables; HR: 1.23, 95% CI: 0.83–1.82 after further adjustment for treatment‐related parameters).

## 4 Discussion

In our cohort of breast cancer patients treated with modern oncological therapy, we found no statistically significant difference in survival outcomes between patients with metBC and a matched cohort of patients with NST. Numerous studies have investigated the outcomes of metBC in comparison to NST, providing valuable insights aimed at enhancing management strategies and evaluating survival outcomes, though the results have often been somewhat contradictory.

Interestingly, the contradictory results among various studies appear to follow a chronological pattern. Specifically, studies involving patient cohorts from the 1990s and early 2000s repeatedly observed worse survival for patients with metBC compared to those with other histologies. In contrast, studies with more recent cohorts, like ours, suggest no difference in prognosis. Polamraju et al. analyzed over 5000 metBC cases from the National Cancer Data Base (NCDB) between 2004 and 2013 and found that metBC had worse overall survival (OS) than triple‐negative breast cancer [1]. Similarly, Ong et al. compared 2451 metBC cases with 568,057 NST cases from 2010 to 2014 in the NCDB, concluding that metBC had poorer OS [7]. However, the median follow‐up was only about 37 months, and unlike our study, no matching on key variables was performed to create a comparable cohort within the NST group. Nelson et al. examined 966 metBC patients against 1932 NST patients from 2001 to 2011 using SEER (Surveillance, Epidemiology, and End Results) data, revealing significantly poorer 5‐year survival rates for metBC patients [3]. Overall, these studies highlight the challenges associated with managing metBC and the limitations of historical data regarding contemporary treatment options. It could be argued that these studies are outdated, as the patients may not have had access to modern oncological therapies, including sequential and/or dose dense chemotherapy [10, 11], modern radiotherapy [12], and anti‐HER2 therapies for the small group of HER2‐positive patients [13]. Interestingly, newer treatment strategies, such as checkpoint inhibitors in the neoadjuvant and adjuvant settings for TNBC, and PARP inhibitors as adjuvant therapy for gBRCA mutation carriers, have shown promising preliminary results in patients with metBC as well [14, 15]. Therefore, contemporary cohorts including patients treated with these modern therapies may be valuable for further assessing the prognosis of metBC in the modern era.

In our matched cohort analysis, we observed that although there was a numerical difference in survival rates favoring NST, there were no statistically significant differences. This aligns with the findings of Lee et al., who matched 120 met BC patients with 478 NST patients in a 1:4 ratio from a single institution in Korea, over a long period (1994 to 2019) [4]. Following propensity score matching, their OS analysis indicated no significant difference between the metBC and NST groups. Similarly, Narayan et al. found only a numerical difference in OS between metBC and NST patients, which did not reach statistical significance [6]. Zhang et al. analyzed SEER data from 2010 to 2017, comparing 841 metBC patients to a matched cohort of NST patients at a 1:4 ratio [5]. They focused on the impact of chemotherapy in any setting on survival, finding that metBC patients who received adjuvant chemotherapy or responded to neoadjuvant chemotherapy had survival outcomes similar to NST patients. However, metBC patients who did not respond to neoadjuvant chemotherapy had poorer BCSS and OS compared to NST patients. These more contemporary studies support our findings.

However, our findings should be interpreted with caution given the study's design. First, the retrospective nature of our cohort introduces potential bias and confounding factors that may have obscured certain associations in the results. To mitigate this, we employed a 2:1 matching process to create a comparison cohort closely resembling the metaplastic group. A central assessment of pathology results was not conducted in this study, which may introduce variability in diagnosing of metBC across different institutions. Additionally, information on histological subtypes of metBC was not available; this may have influenced our prognostic results, as subtype has been shown to affect outcomes [16]. Another limitation is the relatively small sample size (*N* = 127) which may limit the ability to detect statistically significant results. Additionally, our study had a relatively short follow‐up period, which may limit the clinical interpretation of our results, as long‐term outcomes are essential for understanding the impact of interventions in metaplastic breast cancer. Our study cohort also primarily consisted of patients with stage I–II disease, which reflects the Swedish breast cancer population due to a well‐organized national screening program with high coverage for women aged 40–74 years. This may limit the generalizability of our results to patients with more advanced disease, such as stage III. Lastly, when interpreting our findings, it is important to acknowledge that the definition of TNBC in our study included ER‐low tumors, although ER status does not appear to substantially influence prognosis within metaplastic breast cancer [17].

In summary, our study results suggest that appropriately treated patients with metBC may have comparable survival outcomes to those with NST. While acknowledging the limitations associated with the study's retrospective design and the relatively small patient cohort, further studies involving larger patient cohorts and detailed information on newer treatment strategies are needed to elucidate the current clinical implication of metBC.

## Author Contributions

Marie Bergman: conceptualization; investigation; funding acquisition; writing – original draft; methodology; validation; writing – review and editing; data curation; project administration. Aglaia Schiza: writing – original draft; writing – review and editing; conceptualization. Ceren Boyaci: writing – review and editing; validation. Balazs Acs: writing – review and editing; validation. Alexios Matikas: writing – review and editing; conceptualization; investigation; data curation. Johan Hartman: writing – review and editing; validation; supervision. Antonis Valachis: conceptualization; investigation; funding acquisition; methodology; validation; visualization; writing – review and editing; formal analysis; project administration; data curation; supervision.

## Funding

This study was supported by the Regional Research Council in Mid Sweden (Regionala Forskningsrådet Region Mellansverige) and the Centre for Clinical Research Värmland (Centrum för klinisk forskning Värmland).

## Ethics Statement

The study was approved by the Swedish Ethical Review Authority (*Etikprövningsmyndigheten*) and conducted in accordance with the Declaration of Helsinki. Ethical approval was granted under the following reference numbers:

- 2019–01834 (main application, approved on 2019‐05‐31)
- 2020–01345 (amendment, approved on 2020‐04‐15)
- 2020–04849 (amendment, approved on 2020‐09‐14)
- 2022–03161‐02 (amendment, approved on 2022‐06‐18)

As the study was retrospective in design, informed consent was not required according to the ethical approvals.

## Conflicts of Interest

The authors declare no conflicts of interest.

## Supporting information


**FIGURE S1:** Flowchart of patient selection.

## Data Availability

The data that support the findings of this study are available on request from the corresponding author, M.B. The data are not publicly available due to restrictions related to privacy and ethical considerations.
