# Supplementary material for: Prognosis of Metaplastic Breast Cancer: A Population‐Based Matched Cohort Study
Source: Cancer Med. 2026 Jan 28;15(2):e71570. doi: 10.1002/cam4.71570 (PMC12848589; doi:10.1002/cam4.71570)
Supplement: Supplementary file 1 — FIGURE S1: Flowchart of patient selection. [file CAM4-15-e71570-s001.docx]

**Supplementary material for Prognosis of metaplastic breast cancer: a population-based matched cohort study**

Supplementary Figure 1. Flowchart of patient selection

Excluded (n = 188)

Lack of information in matching variables (n = 158)

Lack of information in outcome (n = 30)

**Swedish Quality Register for breast cancer**

Matched patients with invasive ductal breast cancer

N = 245

**Linkage between Cancer Register and Swedish Quality Register for breast cancer**

Patients with metaplastic breast cancer eligible for matching

N = 127

**Swedish Cancer Register**

Patients with diagnosis of metaplastic breast cancer between 2008 – 2018

N = 315
